# Immune-related gene *IL17RA* as a diagnostic marker in osteoporosis

**DOI:** 10.3389/fgene.2023.1219894

**Published:** 2023-08-04

**Authors:** Ya-Jun Deng, Zhi Li, Bo Wang, Jie Li, Jun Ma, Xiong Xue, Xin Tian, Quan-Cheng Liu, Ying Zhang, Bin Yuan

**Affiliations:** Department of Spine Surgery, Xi’an Daxing Hospital, Yanan University, Xi’an, China

**Keywords:** IL17RA, osteoporosis, gene expression omnibus, DEIRGs, diagnostic markers

## Abstract

**Objectives:** Bone immune disorders are major contributors to osteoporosis development. This study aims to identify potential diagnostic markers and molecular targets for osteoporosis treatment from an immunological perspective.

**Method:** We downloaded dataset GSE56116 from the Gene Expression Omnibus database, and identified differentially expressed genes (DEGs) between normal and osteoporosis groups. Subsequently, differentially expressed immune-related genes (DEIRGs) were identified, and a functional enrichment analysis was performed. A protein-protein interaction network was also constructed based on data from STRING database to identify hub genes. Following external validation using an additional dataset (GSE35959), effective biomarkers were confirmed using RT-qPCR and immunohistochemical (IHC) staining. ROC curves were constructed to validate the diagnostic values of the identified biomarkers. Finally, a ceRNA and a transcription factor network was constructed, and a Gene Ontology and Kyoto Encyclopedia of Genes and Genomes enrichment analysis was performed to explore the biological functions of these diagnostic markers.

**Results:** In total, 307 and 31 DEGs and DEIRGs were identified, respectively. The enrichment analysis revealed that the DEIRGs are mainly associated with Gene Ontology terms of positive regulation of MAPK cascade, granulocyte chemotaxis, and cytokine receptor. protein–protein interaction network analysis revealed 10 hub genes: *FGF8*, *KL*, *CCL3*, *FGF4*, *IL9*, *FGF9*, *BMP7*, *IL17RA*, *IL12RB2*, *CD40LG*. The expression level of *IL17RA* was also found to be significantly high. RT-qPCR and immunohistochemical results showed that the expression of *IL17RA* was significantly higher in osteoporosis patients compared to the normal group, as evidenced by the area under the curve Area Under Curve of 0.802. Then, we constructed *NEAT1*-hsa-miR-128-3p-*IL17RA*, and *SNHG1*-hsa-miR-128-3p-*IL17RA* ceRNA networks in addition to *ERF*-*IL17RA*, *IRF8*-*IL17RA*, *POLR2A*-*IL17RA* and *ERG*-*IL17RA* transcriptional networks. Finally, functional enrichment analysis revealed that *IL17RA* was involved in the development and progression of osteoporosis by regulating local immune and inflammatory processes in bone tissue.

**Conclusion:** This study identifies the immune-related gene *IL17RA* as a diagnostic marker of osteoporosis from an immunological perspective, and provides insight into its biological function.

## 1 Introduction

Osteoporosis is the most common metabolic bone disease, characterized by reduced bone density and deterioration of bone tissue microarchitecture, increases bone fragility and the risk of fractures, leading to significant mortality (Chandra and Rajawat, 2021). Osteoporosis primarily affects postmenopausal women and men over the age of 50 (Borgström et al., 2020). The pathogenesis of osteoporosis exhibits noteworthy disparities between genders. In women, age-related bone loss and decreased estrogen secretion after menopause are the primary contributors to this condition (Shih et al., 2019). Estrogen plays a pivotal role in augmenting bone cell activity, inhibiting bone resorption, and averting calcium loss from bones. Moreover, estrogen restrains osteoclast formation and induces apoptosis in these cells, thereby curtailing bone resorption. In the absence of sufficient estrogen levels, osteoclast function heightens, leading to accelerated bone loss and the eventual onset of osteoporosis (Zhou et al., 2001). In men, the principal causes of osteoporosis include advancing age, prolonged glucocorticoid use, and declining testosterone levels (Vilaca et al., 2022). With age, inadequate testosterone levels impede the proliferation and differentiation of osteoblasts while intensifying osteoclast activity. Consequently, bone resorption escalates, resulting in subsequent loss of bone mass (Diab and Watts, 2021). It is evident that testosterone plays a pivotal role in the development of osteoporosis among elderly men.

Dual-energy x-ray absorptiometry (DXA) is considered the gold standard for diagnosing osteoporosis (Carey et al., 2022). Nonetheless, it has limitations when it comes to detecting early-stage bone loss. Early diagnosis and timely intervention are beneficial for preventing the development of osteoporosis (Sakka, 2022). In recent years, numerous studies have demonstrated that *CUL1*, *PTEN*, *STAT1*, *MAPKAPK2*, *RARRES2*, *FLNA*, *STXBP2*, miR-340-5p, and miR-506-3p could potentially serve as biomarkers for osteoporosis (Wang et al., 2022; Zhao Y. et al., 2022; Lu et al., 2023; Yuxuan et al., 2023). However, the majority of these molecular markers have not yet been validated using clinical samples. Thus, their potential for clinical applications remain limited. Therefore, there is still a need to find effective biomarkers for osteoporosis.

Osteoclasts, originating from hematopoietic cells of the myeloid lineage, play a crucial role in bone resorption (Thiolat et al., 2014). These cells undergo differentiation from osteoclast precursors when stimulated by M-CSF and RANKL (Zheng et al., 2014). Osteoblasts play a fundamental role in the synthesis of mineralized bone and are derived from a mesenchymal progenitor cell (Debnath et al., 2018). Multiple immune cells are involved in the regulation of osteoclast and osteoblast homeostasis. Th17 cells induce osteoclastogenesis by IL-17, Th1 cells activate osteoclast function through TNF-α (Adamopoulos and Bowman, 2008; Liu et al., 2011). Conversely, Sato et al. (2006) demonstrated that Th2 cells can impede osteoclast formation via IL-4. DCs enhance osteoclast activity by interacting with T cells through the RANK-RANKL signaling pathway (Santiago-Schwarz et al., 2001). Rivollier et al. (2004) revealed that DCs can transdifferentiate into osteoclasts *in vitro* in the presence of M-CSF and RANKL. Furthermore, B cells secrete RANKL, promoting osteoclast function (Kanematsu et al., 2000). Conversely, ILC2 cells suppress osteoclast formation through the release of IL-4 and IL-13 (Omata et al., 2020). Treg cells can inhibit monocyte differentiation into osteoclasts (Luo et al., 2011). Neutrophils hinder bone formation by affecting osteoblast function (Brunetti et al., 2013). M2 macrophages promote osteoblast differentiation (Vi et al., 2015). Conversely, M1 macrophages leads to bone resorption by increasing osteoclast activity and suppressing osteoblast-mediated bone formation (Bastian et al., 2011). *In vitro* studies have demonstrated the direct enhancement of osteoblast function by Treg cells (Lei et al., 2015). Additionally, B cells activate NF-κB signaling pathways to inhibit the differentiation of mesenchymal precursor cells into osteoblasts (Sun et al., 2018). Therefore, exploring the molecular mechanisms of osteoporosis from an immune perspective and developing new targets for immunotherapy is of great relevance for osteoporosis treatment.

Here, we performed a differential gene expression analysis on an osteoporosis microarray dataset downloaded from the Gene Expression Omnibus (GEO) database, and identified the intersection of differentially expressed genes (DEGs) with immune-related genes (IRGs) to determined differentially expressed immune-related genes (DEIRGs). Then, we constructed a protein–protein interaction (PPI) network to identify hub genes, and finally determined the immune-related gene *IL17RA* as a potential biomarker for osteoporosis after validating it in another dataset (GSE35959). RT-qPCR and immunohistochemical (IHC) staining were performed, and then ROC curves were constructed to verify its diagnostic value. In addition, we also explored the biological function of IL17RA by constructing ceRNA and transcription factor (TF) networks in addition to a Gene Ontology (GO) and Kyoto Encyclopedia of Genes and Genomes (KEGG) enrichment analysis to further elucidate the molecular mechanisms of osteoporosis in which *IL17RA* is involved.

## 2 Materials and methods

### 2.1 Microarray data

**FIGURE 1:**
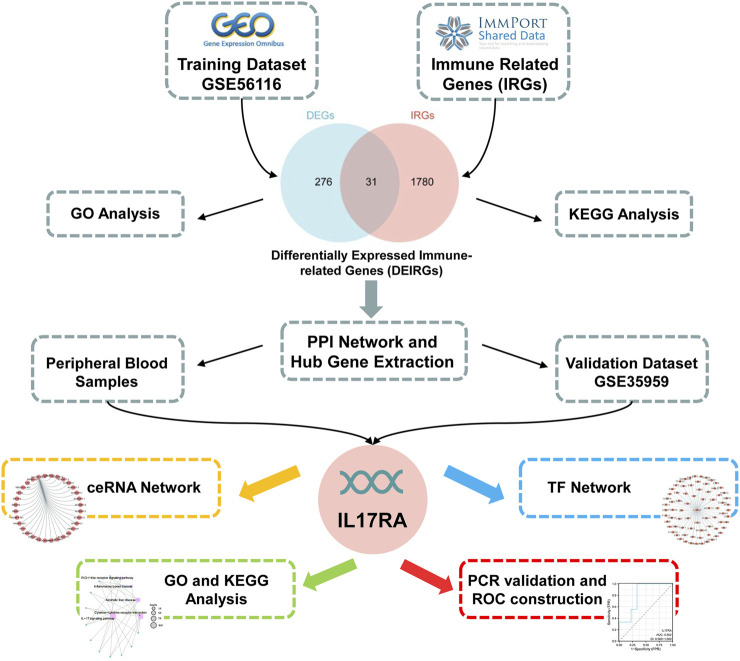
Flow chart of the study.

mRNA [GSE56116, https://www.ncbi.nlm.nih.gov/geo/query/acc.cgi?acc=GSE56116, GSE35959 (Benisch et al., 2012)], and miRNA microarray datasets [GSE201543 (Zhao S.-L. et al., 2022)] were downloaded from GEO (http://www.ncbi.nlm.nih.gov/geo/) by GEOquery package (Davis and Meltzer, 2007). GSE56116 was obtained from a GPL4133 Agilent-014850 Whole Human Genome Microarray 4 × 44 K G4112F. GSE35959 was obtained from a GPL570 (HG-U133_Plus_2) Affymetrix Human Genome U133 Plus 2.0 Array and GSE201543 was obtained from a GPL20712 Agilent-070156 Human miRNA (miRNA version). The probes were labeled with gene symbols, then multiple probes corresponding to the same gene were randomly selected to remove duplicates, and finally the gene expression matrix was obtained. GSE56116 contained 3 normal (healthy control) and 10 osteoporosis samples (4 kidney Yin deficiency, 3 kidney Yang deficiency, 3 non-kidney deficiency). GSE35959 contained 14 normal and 5 osteoporosis samples. GSE201543 contained 4 normal and 6 osteoporosis samples. GSE56116 (3 non-kidney deficiency osteoporosis samples and 3 normal samples), GSE35959, and GSE201543 were used as the training, validation, and miRNA validation datasets, respectively. Data of IRGs was downloaded from ImmPort database (https://www.immport.org/shared/), and finally 2,499 immune-related genes were obtained (Supplementary Table S1). The flow chart followed this study is shown in Figure 1.

### 2.2 Identification of DEIRGs

The data set was normalized using the normalizeBetweenArrays function of the limma package (Ritchie et al., 2015). The sample correction is visualized by box plots, and the clustering between sample groups was visualized using PCA plots. Screen for DEGs between patients and controls using the limma package, and *p* value of lower than 0.05 and [log2 fold change (FC)] equal to or higher than 1 were set as the threshold values for DEG identification. After that, the intersection of DEGs and IRGs was determined to obtain list of DEIRGs. The results were visualized using the ggplot2 (Wickham, 2009) and the VennDiagram packages (Chen and Boutros, 2011). The results of DEGs and DEIRGs were visualized using the ggplot2 package for volcano plots and the Complex Heatmap package (Gu et al., 2016) for heat maps.

### 2.3 GO and KEGG enrichment analyses of DEIRGs

GO and KEGG functional enrichment analysis of DEIRGs were conducted using the cluster Profiler package (Yu et al., 2012). The results were visualized using the ggplot2 package. The human genome was used as a background reference, and a p.adj of lower than 0.05 was set as cut-off.

### 2.4 Construction of PPI network and selection of hub genes

The PPI network of DEIRGs was constructed using the STRING database (https://string-db.org/) (Szklarczyk et al., 2019). Interaction scores higher than 0.4 were considered significant. The results were visualized using Cytoscape software (Version: 3.9.1). Top 10 genes were determined as hub genes using the cytoHubba plugin and on the maximum correlation criterion algorithm.

### 2.5 External validation of hub genes

To identify effective biomarkers of osteoporosis, differences in hub gene expression levels between the osteoporosis and the normal groups were validated using another dataset (GSE35959).

### 2.6 Construction of ceRNA network

MiRNAs were predicted using four different databases [miRDB (https://mirdb.org/mirdb/index.html), TargetScanHuman (https://www.targetscan.org/vert_80/), TarBase (https://dianalab.e-ce.uth.gr/html/diana/web/index.php?r=tarbasev8) and miRWalk (http://mirwalk.umm.uni-heidelberg.de/)]. Furthermore, lncRNA-miRNA relationships of predicted hub genes-associated miRNAs were obtained by overlapping results from starBase (http://starbase.sysu.edu.cn/) and DIANA-LncBase v3 (https://diana.e-ce.uth.gr/lncbasev3). The overlap was visualized by ggplot2 and VennDiagram packages. Finally, a competitive endogenous RNA (ceRNA) network regulating hub genes was constructed by using Cytoscape software (Version: 3.9.1).

### 2.7 Construction of TF network

Prediction of hub genes and their TFs were performed by TF-Marker (http://bio.liclab.net/TF-Marker/) and GRNdb (http://www.grndb.com/), the overlap was visualized by ggplot2 and VennDiagram packages. A TF network regulating hub genes was constructed by using igraph package (Csardi and Nepusz, 2006).

### 2.8 Study subjects

Peripheral blood samples were obtained from nine patients with osteoporosis and nine healthy adults who were hospitalized in the Department of Spine Surgery at Xi’an Daxing Hospital, affiliated with Yan’an University, and underwent BMD testing between January 2023 and April 2023 (Table 1). Those with a history of long-term use of drugs affecting bone metabolism, endocrine system disorders, spinal tumors or spinal tuberculosis were not included in this study. Bone tissue samples were obtained from twelve patients with osteoporotic compression fractures who were hospitalized for vertebroplasty surgery, with mild osteoporosis (−3. 5 < T-score ≤ −2. 5) and severe osteoporosis (T-score ≤ −3. 5), six in each group (Table 2). All patients underwent MRI and DXA of the spine, which confirmed the presence of fresh fractures and osteoporosis. Inclusion criteria were as follows: 1) age ≥50 years, BMD T-score ≤ −2.5; 2) vertebral fragility fracture, biopsy routinely performed during vertebroplasty. Exclusion criteria were as follows: previous long-term use of drugs affecting bone metabolism, presence of endocrine system diseases, spinal tumors and spinal tuberculosis. The diagnosis of osteoporosis was confirmed based on the classifcation criteria of the World Health Organization (WHO) based on T-score of BMD testing (Yoshimura et al., 2022): T-score ≥ −1.0 was considered normal bone mass, −2.5 < T-score < −1.0 was considered decreased bone mass, and T-score ≤ −2.5 was considered osteoporosis. All included subjects were informed of the medical record review and study design and signed consent documents before data collection. The Ethics Committee of the Xi’an Daxing Hospital, affiliated with Yan’an University approved and reviewed the study protocol.

**TABLE 1:** Study subject demographics of peripheral blood.

Characteristics	Normal	Osteoporosis	*p* Value
n	9	9	
Gender, n (%)			1.000
Female	5 (27.8%)	5 (27.8%)	
Male	4 (22.2%)	4 (22.2%)	
Age (year)	56.11 ± 9.35	63 ± 11.12	0.174
BMI (kg/m^2^)	24.63 ± 3.33	23.65 ± 2.37	0.482
BMD (g/cm^2^)	0.98 ± 0.13	0.73 ± 0.08	< 0.001
T-score	−0.87 ± 1.13	−3.07 ± 0.58	< 0.001

**TABLE 2:** Study subject demographics of bone tissue.

Characteristics	Mild osteoporosis	Severe osteoporosis	*p* Value
n	6	6	
Gender, n (%)			1.000
Female	3 (25%)	4 (33.3%)	
Male	3 (25%)	2 (16.7%)	
Age (year)	65.33 ± 10.03	66.83 ± 12.62	0.824
BMI (kg/m^2^)	22.39 ± 1.45	20.79 ± 1.28	0.070
BMD (g/cm^2^)	0.73 ± 0.05	0.58 ± 0.08	0.002
T-score	−2.78 ± 0.15	−3.95 ± 0.53	0.002

### 2.9 Peripheral blood collection

Five milliliters peripheral blood samples were collected the morning following an overnight fast. The serum was obtained following centrifugation (3,000 r/min, 5 min) of blood samples and submitted for bone metabolism marker detection. Cell sediment was collected for RNA extraction. All samples were frozen at −80°C until analysis.

### 2.10 Bone tissue sample acquisition

The patient was placed in the prone position, and a 0.5–1 cm piece of cancellous bone tissue was drilled using a 14G bone biopsy ring perched on the fracture area within the vertebral body under local anesthesia via the arch root approach. The bone tissue was fixed in 10% neutral-buffered formalin for 1 week, followed by routine decalcification, dehydration, and paraffin embedding for subsequent studies.

### 2.11 BMD measurements

BMD measurements of the lumbar spine were performed using DXA (QDR X-Ray Bone Densitometer, Hologic, United States). All data were measured by the same group of imaging physicians in strict accordance with the specifications for measuring BMD by DXA.

### 2.12 RT-qPCR analysis

Total RNA was extracted using RNA Extraction Solution (G3013, Servicebio, Wuhan, China), and RNA concentration and purity were measured by Nanodrop 2000 spectrophotometer (Thermo Scientific, Waltham, United States). The RNA samples were reverse transcribed into cDNA using a reverse transcription kit (G3337, Servicebio, Wuhan, china), and the cDNA was used as a template to amplify the IL17RA gene. The reaction was performed via 40 amplification cycles using the following protocol: Denaturation at 95°C for 30 s, annealing at 60°C for 30 s, extension at 72°C for 60 s. Samples were analyzed in triplicate, the mRNA expression levels of IL17RA was calculated by the 2^−ΔΔCT^ method, and GAPDH was used as internal reference. The sequences of the primers are listed in Table3.

**TABLE 3:** Primer sequences used for RT-qPCR.

Gene	Forward primer (5′–3′)	Reverse primer (5′–3′)
IL17RA	CCA​ACA​TCA​CCG​TGG​AGA​CC	GTG​GCG​ACA​GCA​CCC​TTT​AA
GAPDH	GGA​AGC​TTG​TCA​TCA​ATG​GAA​ATC	TGA​TGA​CCC​TTT​TGG​CTC​CC

### 2.13 HE and IHC staining

The wax blocks were placed in a paraffin slicer for continuous sectioning, with each section having 4 μm thickness. HE staining was performed using HE staining solution (G1003, Servicebio, Wuhan, China). For IHC, paraffin tissue sections were deparaffinized with xylene and rehydrated with an alcohol gradient and water. Sections were incubated with primary antibodies IL17RA (Catalogue number: DF3602, diluted 1:100, Affinity), at room temperature for 1 h and biotin-labelled secondary antibodies for 30 min, and then stained with DAB peroxidase substrate kit (G1212, Servicebio, Wuhan, China). Finally, washed with water and counterstained with haematoxylin. The results were observed and photographed by an microscope (Eclipse C1, Nikon, Japan).

### 2.14 Statistical analysis

All data processing and analysis were conducted using R software (version 4.2.1). RT-qPCR were repeated three times, and data were represented as the mean ± SD. Normality was tested using the Shapiro-Wilk normality test and chi-squaredness was tested using Levene’s test. Student’s t-test or Wilcoxon rank sum test was used to determine the significance of difference between two groups. Correlation coefficients were calculated using Spearman correlation analysis. ROCs were used to evaluate AUCs and predictive abilities. A *p* value lower than 0.05 was considered statistically significant.

## 3 Results

### 3.1 Identification of DEIRGs

**FIGURE 2:**
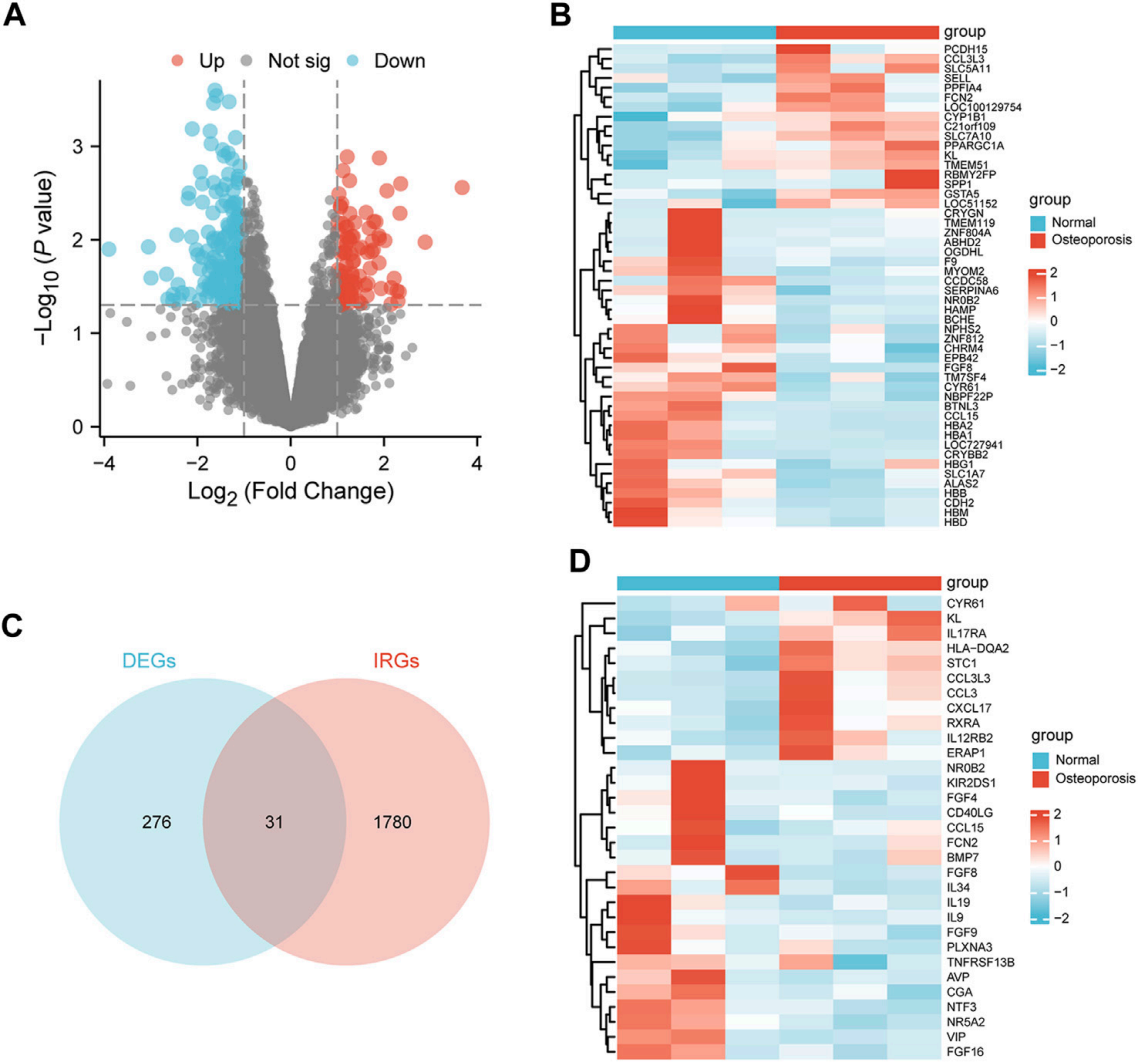
Identification of DEIRGs. **(A)** Volcano plot, **(B)** heatmap of DEGs between the osteoporosis and normal samples. **(C)** Venn diagram of overlapping genes between the DEGs and IRGs. **(D)** Heatmap of DEIRGs.

The median, upper and lower quartiles, maximum and minimum values of each sample gene were significantly close to each other upon normalization of GSE56116 data (Supplementary Figure S1A, B). However, PCA revealed that the centers of the osteoporosis group were farther apart than those of the control group, indicating significant differences in gene expression between the two groups (Supplementary Figure S1C, D). Using a *p* value of lower than 0.05 and a [log2 fold change (FC)] equal to or higher than 1 as the threshold levels, we identified 307 DEGs, including 94 and 213 significantly up- and down-regulated genes, respectively (Supplementary Table S2). Figures 2A, B show results in the form of volcano plots and heat maps (Figures 2A, B). The intersection of DEGs and IRGs included 31 genes (DEIRGs) (Figure 2C; Supplementary Table S3), including 11 and 20 up- and down-regulated genes, respectively (Figure 2D).

### 3.2 Functional enrichment analyses of DEIRGs

**FIGURE 3:**
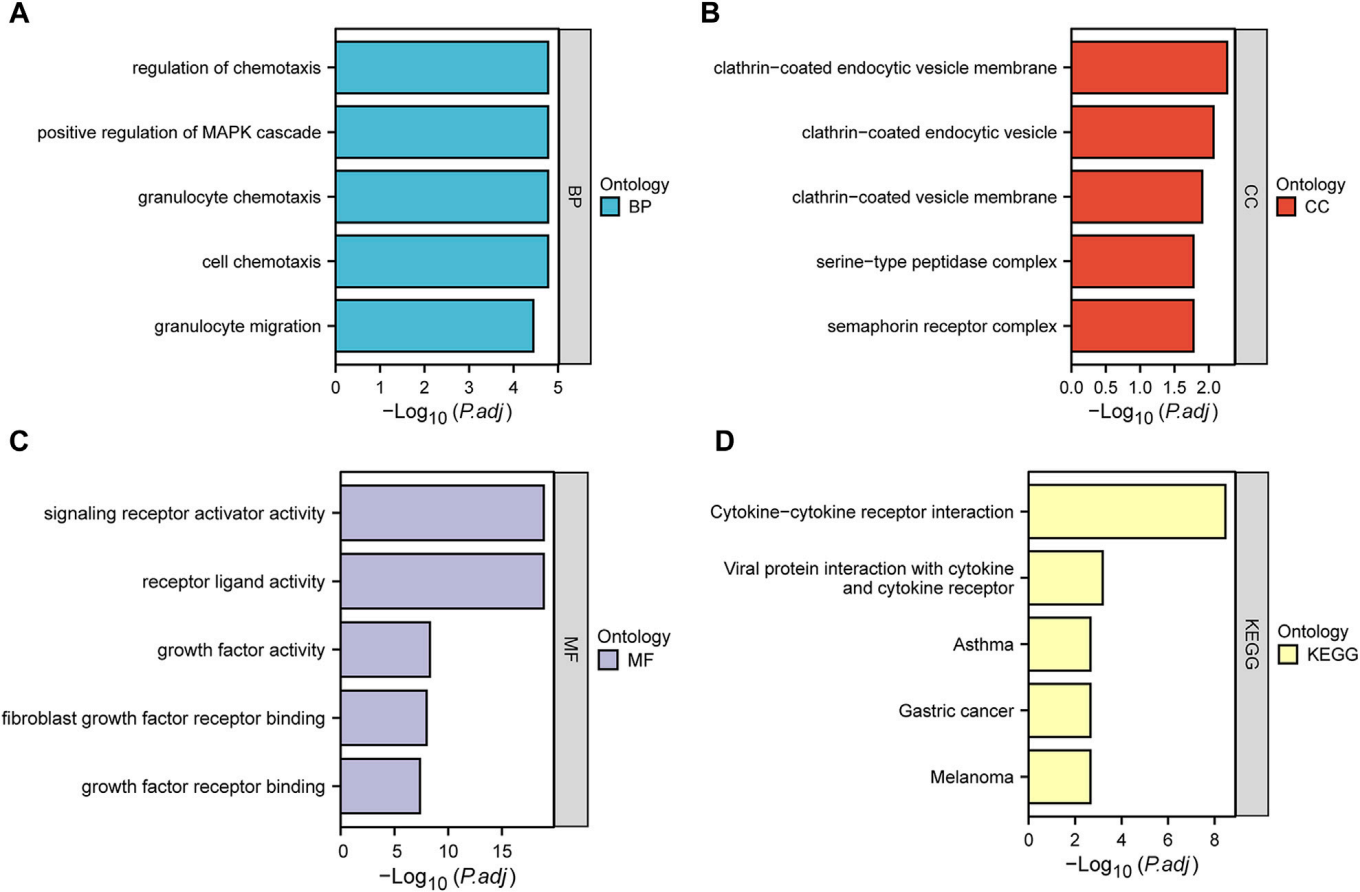
Bar plots of 31 DEIRGs-enriched GO terms and KEGG pathways. **(A–D)** represent BP, CC, MF, and KEGG, respectively.

We performed GO and KEGG enrichment analysis to investigate the functions of DEIRGs. In the GO analysis, biological processes (BPs), cell components (CCs), and molecular functions (MFs) were distinguished. The BPs included regulation of chemotaxis, positive regulation of MAPK cascade, granulocyte chemotaxis, cell chemotaxis and granulocyte migration. CCs included clathrin−coated endocytic vesicle membrane, clathrin−coated endocytic vesicle, clathrin−coated vesicle membrane, serine−type peptidase complex and semaphorin receptor complex. Finally, MFs included signaling receptor activator activity, receptor ligand activity, growth factor activity, fibroblast growth factor receptor binding and growth factor receptor binding. KEGG analysis showed that DEIRGs were mainly associated with Cytokine−cytokine receptor interaction, Viral protein interaction with cytokine and cytokine receptor. Figures 3A–D show the top five enrichment items of BP, CC, MF in GO and KEGG analyses.

### 3.3 Construction of the PPI network and identification of hub genes

**FIGURE 4:**
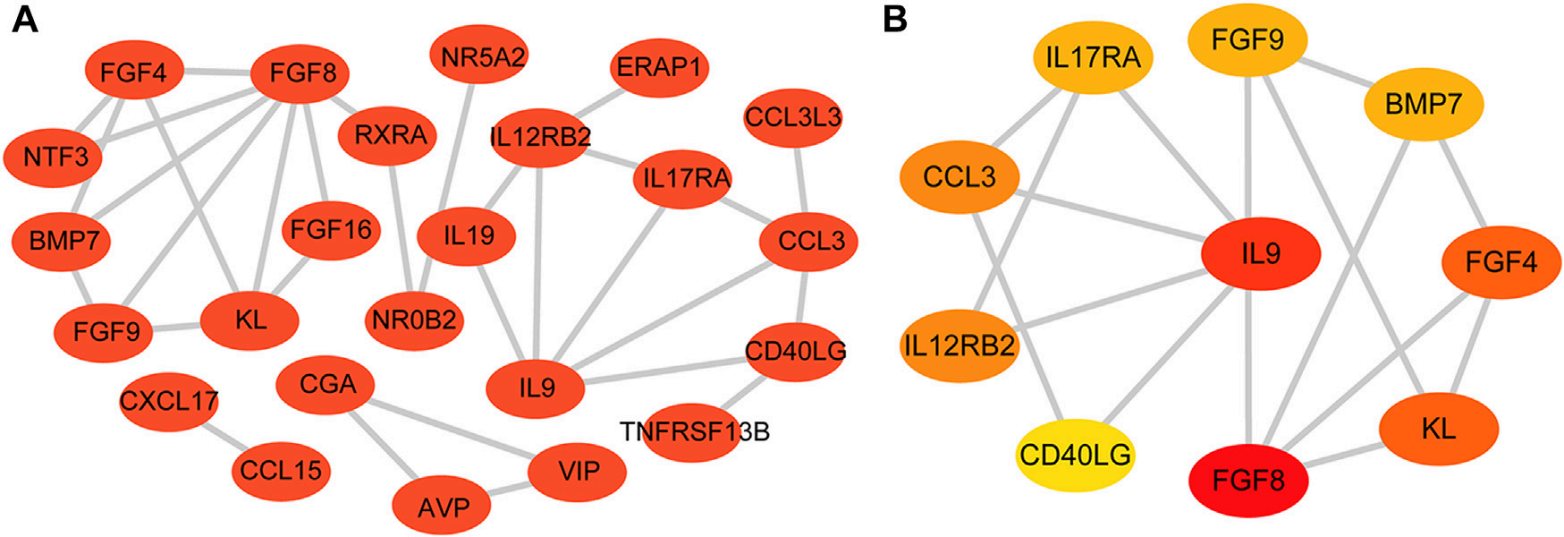
PPI network and hub genes. **(A)** PPI network constructed with the DEIRGs; **(B)** Top 10 hub genes.

The STRING database was used to construct a PPI network of 31 DEIRGs in order to investigate protein-protein interactions. A total of 30 nodes and 31 edges were identified in the PPI network (Figure 4A). The cytohubba plug-in of Cytoscape software was then used to select the top 10 hub genes based on their degree of connectivity (Figure 4B; Table 4).

**TABLE 4:** Top 10 hub genes.

Gene symbol	Entrez id	Full name	logFC	*p*-value
FGF8	2253	Fibroblast growth factor 8	−2.4426	0.008896
KL	9365	Klotho	2.327417	0.036147
CCL3	6348	C-C motif chemokine ligand 3	2.064109	0.002997
FGF4	2249	Fibroblast growth factor 4	−1.60047	0.003061
IL9	3578	Interleukin 9	−1.49079	0.037584
FGF9	2254	Fibroblast growth factor 9	−1.23376	0.024612
BMP7	655	Bone morphogenetic protein 7	−1.23075	0.028784
IL17RA	23765	Interleukin 17 receptor A	1.162541	0.022572
IL12RB2	3595	Interleukin 12 receptor subunit beta 2	1.093632	0.042559
CD40LG	959	CD40 ligand	−1.07323	0.015333

### 3.4 Validation of diagnostic biomarkers

**FIGURE 5:**
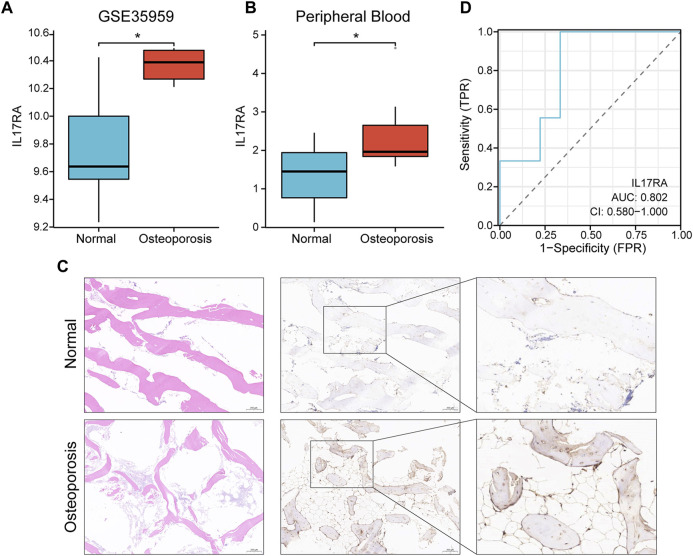
Verification of the diagnostic effectiveness of *IL17RA*. **(A)** *IL17RA* expression in the GSE35959 dataset. **(B)** *IL17RA* expression in peripheral blood samples. **(C)** *IL17RA* expression in bone tissue samples. **(D)** ROC curve.

In the GSE35959 dataset, osteoporotic patients had significantly higher *IL17RA* expression levels than those of patients in the control group (*p* < 0.05) (Figure 5A; Supplementary Figure S2). To confirm the higher expression level of *IL17RA* in osteoporotic patients and its diagnostic performance, we validated this finding using clinical peripheral blood and bone tissue. RT-qPCR results showed that mRNA expression levels of *IL17RA* were significantly higher in peripheral blood of patients with osteoporosis compared to those of patients in the control group (*p* < 0.05) (Figure 5B). The IHC results showed that *IL17RA* expression was higher in the severe osteoporosis group compared to the mild osteoporosis group (Figure 5C). The horizontal and vertical coordinates of the ROC curve indicate sensitivity and specificity, respectively. A larger AUC indicates a more accurate diagnostic model. Accordingly, the AUC was 0.802 (Figure 5D), indicating significant differences between OP and control groups. Hence, *IL17RA* expression level could serve as a good diagnostic biomarker.

### 3.5 Construction of ceRNA network

**FIGURE 6:**
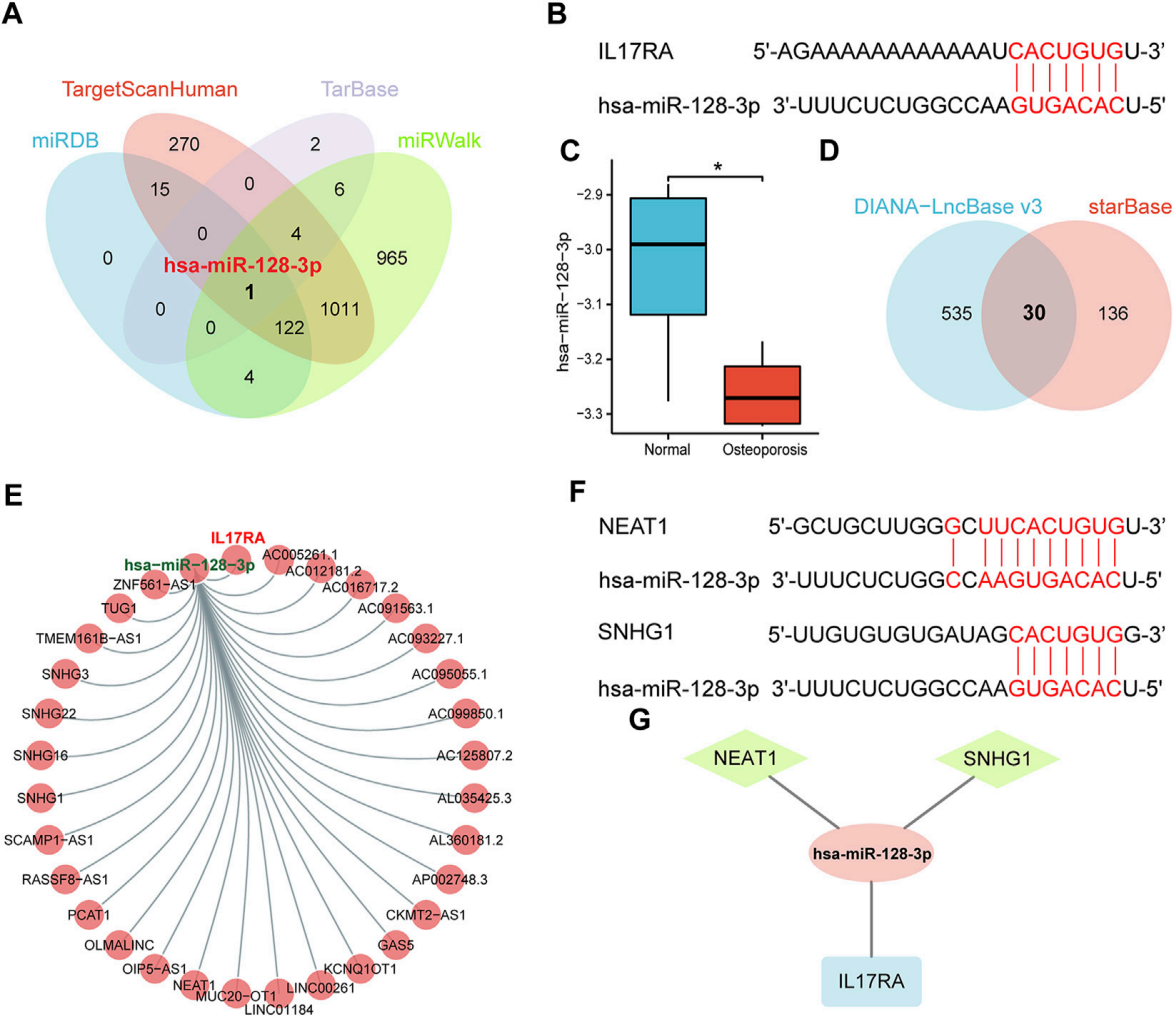
The ceRNA regulatory network of *IL17RA*. **(A)** Prediction of miRNAs targeting *IL17RA* using four different databases. **(B)** Predicted interaction between hsa-miR-128-3p and *IL17RA*. **(C)** Expression of hsa-miR-128-3p in the GSE201543 dataset. **(D)** Prediction of lncRNAs targeting hsa-miR-128-3p using two different databases. **(E)** lncRNA-miRNA-mRNA network of IL17RA. **(F)** Predicted interactions between *NEAT1*, *SNHG1* and hsa-miR-128-3p. **(G)** A ceRNA network consisting of *IL17RA*, hsa-miR-128-3p, *NEAT1* and *SNHG1* in osteoporosis.

We analyzed upstream regulation of *IL17RA,* and screened for miRNAs or lncRNAs targeting *IL17RA*. We identified 142, 1,423, 13, and 2,113 miRNAs possibly targeting *IL17RA* from miRDB, TargetScanHuman, TarBase, and miRWalk databases, respectively (Figure 6A). Consequently, we determined hsa-miR-128-3p to be the most important miRNA regulator by comparing predictions based on each database. Complementary sequences between *IL17RA* and hsa-miR-128-3p are displayed in Figure 6B. We validated hsa-miR-128-3p expression in the GSE201543 dataset, and found that expression level of hsa-miR-128-3p was significantly low in osteoporotic patients (Figure 6C). Next, 30 lncRNAs that could bind to hsa-miR-128-3p were obtained from the overlapping results of DIANA-LncBase v3 and starBase databases (Figure 6D). A lncRNA-miRNA-mRNA network regulating *IL17RA* was constructed, in which lncRNAs competitively bind to miRNAs and attenuate the inhibition of *IL17RA* by miRNAs (Figure 6E). A review of the literature was used to determine these 30 lncRNAs. Our findings indicated that expression levels of *NEAT1* and *SNHG1* were significantly high in osteoporotic patients. Since a significantly high level of *IL17RA* expression and a significantly low level of hsa-miR-128-3p were found in osteoporotic patients, the interactions predicted by the above database (Figure 6F) further led us to hypothesize that *NEAT1* and *SNHG1* bind to hsa-miR-128-3p, and impair the inhibitory effect of hsa-miR-128-3p on *IL17RA* in osteoporosis (Figure 6G).

### 3.6 Transcriptome analysis

**FIGURE 7:**
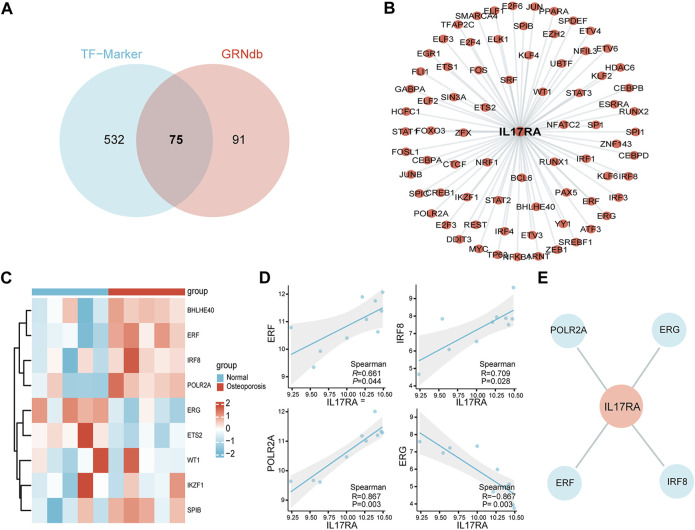
Transcriptional network of *IL17RA*. **(A)** Predicted TFs associated with *IL17RA* based on TF-Marker and GRNdb databases. **(B)** *IL17RA* transcriptional regulatory network. **(C)** Heat map of TFs expression between osteoporosis and normal groups. **(D)** Spearman correlation between *ERF*, *IRF8*, *POLR2A* and *ERG* and *IL17RA*. **(E)** Transcriptional network between *ERF*, *IRF8*, *POLR2A*, *ERG* and *IL17RA* in osteoporosis.

To better understand gene expression upstream of *IL17RA*, we performed a transcriptome analysis. First, we obtained 607 and 166 TFs regulating *IL17RA* from the TF-Marker and GRNdb databases, respectively. A total of 75 TFs were found in both databases (Figure 7A). Using these, an *IL17RA* transcriptional regulatory network was constructed (Figure 7B). We selected nine TFs that showed significant differential expression between the osteoporosis and normal groups (Figure 7C). Among these TFs, *ERF* (*R* = 0.661, *p* = 0.044), *IRF8* (*R* = 0.709, *p* = 0.028), *POLR2A* (*R* = 0.867, *p* = 0.003) and *ERG* (*R* = −0.867, *p* = 0.003) were found to be correlated with *IL17RA* (Figure 7D; Supplementary Figure S3). Based on this, we constructed the osteoporosis *ERF-IL17RA*, *IRF8-IL17RA*, *POLR2A-IL17RA* and *ERG-IL17RA* transcriptional networks (Figure 7E).

### 3.7 GO and KEGG pathway enrichment analysis of diagnostic biomarkers

**FIGURE 8:**
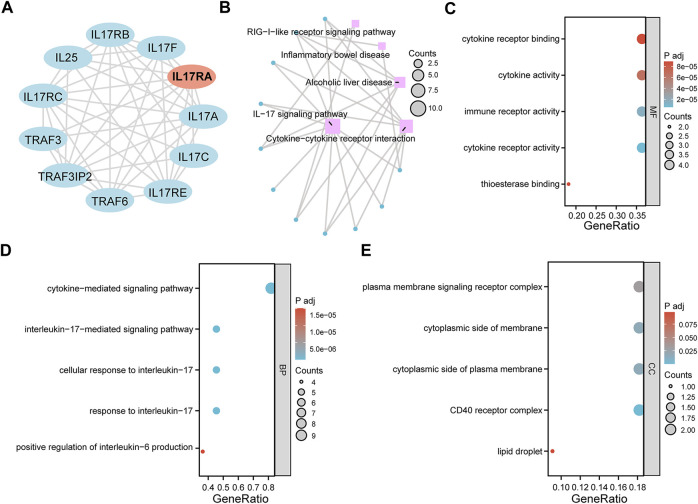
GO and KEGG pathway enrichment analysis. **(A)** An PPI network of *IL17RA* constructed with data from STRING. **(B)** KEGG enrichment analysis network diagram. **(C)** MF **(D)** BP **(E)** CC enrichment analysis bubble diagram.

To investigate the downstream regulatory roles of *IL17RA*, we used the STRING database to predict 10 *IL17RA*-interacting genes (using a confidence score of equal to or higher than 0.4), and constructed a PPI network using Cytoscape (Figure 8A). A total of five KEGG pathways were highlighted by KEGG analysis of *IL17RA* and *IL17RA-*interacting genes: IL−17 signaling pathway, Cytokine−cytokine receptor interaction, alcoholic liver disease, inflammatory bowel disease, and RIG−I−like receptor signaling pathway (Figure 8B). The GO enrichment analysis results indicated that cytokine receptor binding, cytokine activity, immune receptor activity, cytokine receptor activity, and thioesterase binding were the top 5 MF terms (Figure 8C). Cytokine−mediated signaling pathway, interleukin−17−mediated signaling pathway, cellular response to interleukin−17, and response to interleukin−17, positive regulation of interleukin−6 production were the top 5 BP terms (Figure 8D). Finally, plasma membrane signaling receptor complex, cytoplasmic side of membrane, cytoplasmic side of plasma membrane, CD40 receptor complex, and lipid droplet were the top 5 CC terms (Figure 8E).

### 3.8 Gene Set Enrichment Analysis (GSEA)

To investigate the functions of IL17RA in osteoporosis, we conducted Gene Set Enrichment Analysis (GSEA) by stratifying samples based on IL17RA expression. The results enriched several important pathways, including “INTERFERON_ALPHA_RESPONSE,” “INTERFERON_GAMMA_RESPONSE,” “IL6_JAK_STAT3_SIGNALING”, “INFLAMMATORY_RESPONSE”, “REACTIVE_OXYGEN_SPECIES_PATHWAY”, and “TNFA_SIGNALING_VIA_NFKB” (Supplementary Figure S4). These pathways play a critical role in immune response, pro-inflammatory reactions, cytokine signaling, and other vital biological processes. The identification and enrichment of these pathways shed light on the intricate connections between IL17RA and multiple molecular mechanisms involved in maintaining bone health and homeostasis.

## 4 Discussion

Osteoporosis is characterized by reduced bone strength and an increased risk of fracture. It is estimated that more than 200 million people worldwide suffer from osteoporosis, with 30%–50% of women experiencing fractures due to osteoporosis during their lifetime (Rachner et al., 2011). Since osteoporosis patients typically exhibit no obvious clinical symptoms before their first fracture, early diagnosis is crucial for timely intervention and pain relief. Hence, there is an urgent need for effective molecular diagnostic markers. Previous studies suggested that the immune system may play a significant role in osteoporosis development (Sapra et al., 2022; Wang et al., 2022), yet the specific immune targets and molecular mechanisms of osteoporosis remain unknown. Microarray technology has enabled the exploration of genetic alterations in osteoporosis, and has proven effective in identifying novel biomarkers for other diseases. In this study, we used bioinformatics methods to identify diagnostic markers for osteoporosis, and validated their diagnostic value using peripheral blood from osteoporosis patients.

An analysis of transcriptome data from peripheral blood samples of osteoporosis patients and healthy individuals yielded a total of 307 DEGs, including 94 up- and 213 down-regulated genes, respectively. The intersection of DEGs and IRGs yielded a total of 31 DEIRGs, including 11 and 20 up- and down-regulated genes, respectively. GO enrichment analysis of DEIRGs showed that the GO terms were associated with positive regulation of MAPK cascade, granulocyte chemotaxis, growth factor activity, and semaphorin receptor complex. KEGG analysis showed that DEIRGs were mainly associated with Cytokine−cytokine receptor interaction, Viral protein interaction with cytokine and cytokine receptor. These findings suggest that immunomodulation plays a significantly role in the development of osteoporosis. MAPK and innate immune signaling pathways are closely-linked through feedback regulation (Kitajima et al., 2018). Previous studies have reported that the MAPK signaling pathway is involved in the regulation of bone metabolism and osteoclast formation (Meng et al., 2021; Wang et al., 2021). Neutrophil chemokines stimulate the growth and development of osteoblasts and chondrocytes (Mori et al., 1997). Cytokine-cytokine receptor interaction, and viral protein interaction with cytokine suggests significant involvement of the immune system and inflammatory cytokines in the progression of osteoporosis. Inflammatory factors inhibit bone formation in part by suppressing osteoblast differentiation, which includes inhibition of Wnt signaling. In addition, they also promote bone resorption by inducing osteoclast differentiation and bone resorption functions, which in turn disrupt bone homeostasis and contribute to the progression of osteoporosis (Ivashkiv et al., 2011). Hence, dysregulation of the immune system can have a detrimental effect on bone integrity, leading to osteoporosis. Our results are consistent with previous findings.

The PPI network analysis revealed 10 key genes associated with osteoporosis: *FGF8*, *KL*, *CCL3*, *FGF4*, *IL9*, *FGF9*, *BMP7*, *IL17RA*, *IL12RB2*, *CD40LG*. Expression level of *IL17RA* was found to be significantly high in osteoporotic patients upon external dataset validation, suggesting that *IL17RA* may be an effective biomarker for osteoporosis. To further confirm the diagnostic performance of *IL17RA*, we verified *IL17RA* expression by RT-qPCR and IHC, and plotted ROC curves. RT-qPCR results showed that the mRNA expression level of *IL17RA* was significantly higher in peripheral blood of osteoporotic patients compared to that of the control group. IHC results were in line with RT-qPCR. The Area Under Curve (AUC) was 0.802, suggesting a high diagnostic value and potential of *IL17RA* as a diagnostic marker for osteoporosis.

The IL-17 family of inflammatory cytokines has gained attention as major contributors to bone formation and bone resorption. Most IL-17 cytokines act by signaling through the receptor complex of *IL17RA*. *IL17RA* signaling in osteoclast precursors were previously demonstrated to contribute to osteoclast formation and subsequent bone loss. Moreover, *IL17RA* deficiency increases bone mass by decreasing the abundance of osteoclast precursors (Roberts et al., 2022). In addition, IL*17RA* in osteoblasts/osteoclasts mediates parathyroid hormone-induced bone loss and enhances osteoblast RANKL production (Li et al., 2019). These studies are in line with our findings. However, Goswami et al. (2009) used the ovariectomy-induced osteoporosis (OVX) model in *IL17RA* (−/−) mice to assess the role of IL*17A* in estrogen deficiency-induced bone loss. The authors showed that *IL17RA* (−/−) mice were consistently more susceptible to OVX-induced bone loss than controls. *IL17A* inhibits bone resorption-related protease expression and osteoclast differentiation in RAW264.7 cells via *IL17RA* (Kitami et al., 2010). These findings suggests that *IL17RA* signaling plays an osteoprotective role in ovariectomy-induced bone loss. This also shows that the role of *IL17RA* in osteoporosis is still controversial, and an increased sample size is needed for an in-depth analysis.

The concept of CeRNA was introduced in 2011 (Salmena et al., 2011). In the ceRNA network, non-coding RNAs, such as lncRNAs or circRNAs, can compete to bind to miRNAs, and thereby weaken the repression of mRNAs by miRNAs. We identified hsa-miR-128-3p as a key regulatory miRNA for IL17RA in osteoporosis. Previous research has indicated that hsa-miR-128-3p can inhibit osteoblast differentiation of bone marrow mesenchymal stem cells by downregulating *RUNX1*, *YWHAB* and *NTRK2* (Zhang W. et al., 2020). In addition, hsa-miR-128-3p promoted the proliferation, migration and osteoclast differentiation of RAW 264.7 cells and upregulated the osteoclastogenic markers c-Fos, NFATc1 and Ctsk (Zhang et al., 2022a). These findings suggests that hsa-miR-128-3p inhibits osteoblast differentiation and promotes osteoclast formation, which is inconsistent with our findings here. Further studies are needed to explain this paradox, and identify other mechanisms involving has-miR-128-3p in osteoporosis. We hypothesize that *NEAT1* and *SNHG1* target hsa-miR-128-3p. Studies have shown that *NEAT1* promotes the proliferation and differentiation of osteoblasts and, regulates the development and progression of osteoporosis (Zhang Y. et al., 2020; Zhao X. et al., 2022). *SNHG1* expression is up-regulated in OVX mice, which inhibits osteoblast differentiation and angiogenesis while promoting osteoclast formation, leading to osteoporosis (Yu et al., 2021; Yu et al., 2022). *NEAT1* and *SNHG1* are thus promising targets for the treatment of osteoporosis. The above-mentioned findings support the conclusions of our study. We constructed the NEAT1-hsa-miR-128-3p-IL17RA and SNHG1-hsa-miR-128-3p-IL17RA networks to provide a theoretical basis for understanding the molecular mechanisms of *IL17RA* involvement in osteoporosis.

We performed a transcriptional analysis as well. The *ERF* (ETS2 repressor factor) is located on Chromosome 19q13.2, and encodes a transcription factor bound directly by ERK1/2 to regulate the RAS-MEK-ERK signal transduction cascade (von Kriegsheim et al., 2009). A study found that reduced doses of ERF lead to complex cranial suture closure in humans and mice, and highlighted *ERF* as a novel regulator of osteogenic stimulation of RAS-ERK signaling (Sr et al., 2013). IRF8 inhibits osteoclastogenesis, and is involved in the development and progression of osteoporosis (Zhao et al., 2009; Jin et al., 2023). RNA polymerase II subunit A (*POLR2A*) encodes the largest catalytic subunit of the RNA polymerase II complex. Liu et al. (2021) showed that *POLR2A* blocks osteoclastic bone resorption and prevented osteoporosis by interacting with *CREB1*. *ERG* is closely associated with Ewing sarcoma (Dunn et al., 1994), cervical cancer (Zhang Z. et al., 2020) and prostate cancer (Dawoud et al., 2021). However, its role in bone metabolism remains unexplored. The TF network constructed here provides a clear direction to better understand the upstream transcriptional mechanism of *IL17RA*. To further investigate the downstream regulatory role of *IL17RA*, we also performed a functional enrichment analysis of *IL17RA* and its interacting genes. Accordingly, *IL17RA* may be involved in the development and progression of osteoporosis by regulating local immune and inflammatory processes in bone tissue.

There were also some limitations in this study. First, the sample size in the dataset selected for this study was small. Although we standardized the raw data, a larger sample size and a higher quality dataset are still needed to verify the reliability of the results. Secondly, although we validated the diagnostic value of *IL17RA* using patients’ peripheral blood samples and bone tissues, the sample size of this study was also limited, and the clinical translational value of IL17RA needs to be validated in a larger number of clinical osteoporosis samples. Finally, a more comprehensive study on molecular biological mechanisms involving *IL17RA* on both cellular and animal levels is needed.

In conclusion, we identified the immune-related gene *IL17RA* as a diagnostic marker of osteoporosis by elucidating its biological function within the immune system. Our findings may provide with a potential immune molecular target for the early diagnosis and treatment of osteoporosis.

## Data Availability

The datasets presented in this study can be found in online repositories. The names of the repository/repositories and accession number(s) can be found in the article/Supplementary Material.
