# Supplementary material for: Immune-related gene IL17RA as a diagnostic marker in osteoporosis
Source: Front Genet. 2023 Aug 4;14:1219894. doi: 10.3389/fgene.2023.1219894 (PMC10436292; doi:10.3389/fgene.2023.1219894)
Supplement: Supplementary file 4 [file Table4.DOCX]

Supplementary Figures

Immune-related gene *IL17RA* as a diagnostic marker in osteoporosis

**Ya-jun Deng, Zhi Li, Bo Wang, Jie Li, Jun Ma, Xiong Xue, Xin Tian, Quan-cheng Liu, Ying Zhang, Bin Yuan***

*** Correspondence:** Bin Yuan: yuanbin8210@163.com


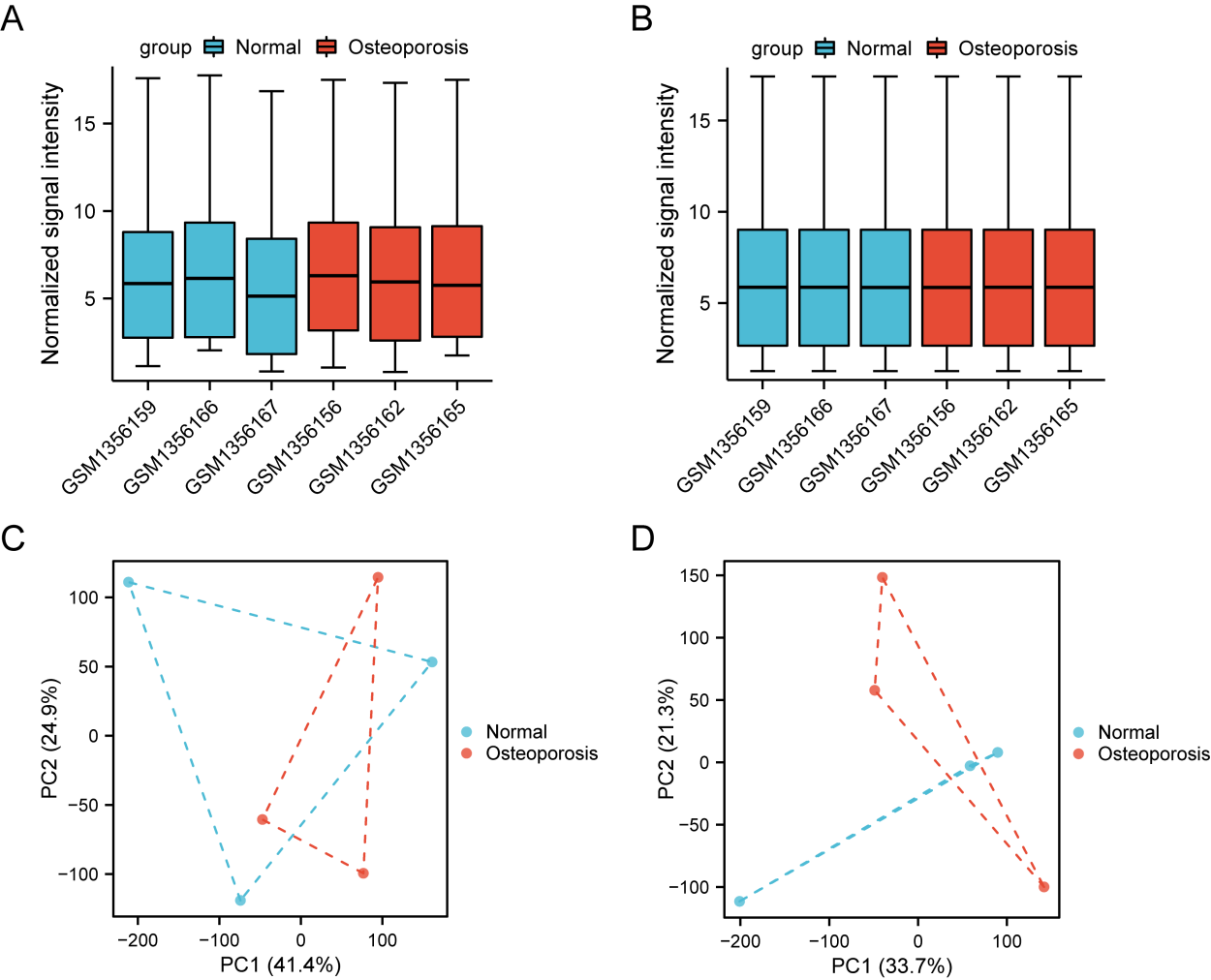


**Supplementary** **figure 1** Box plots and PCA of GSE56116 datasets.


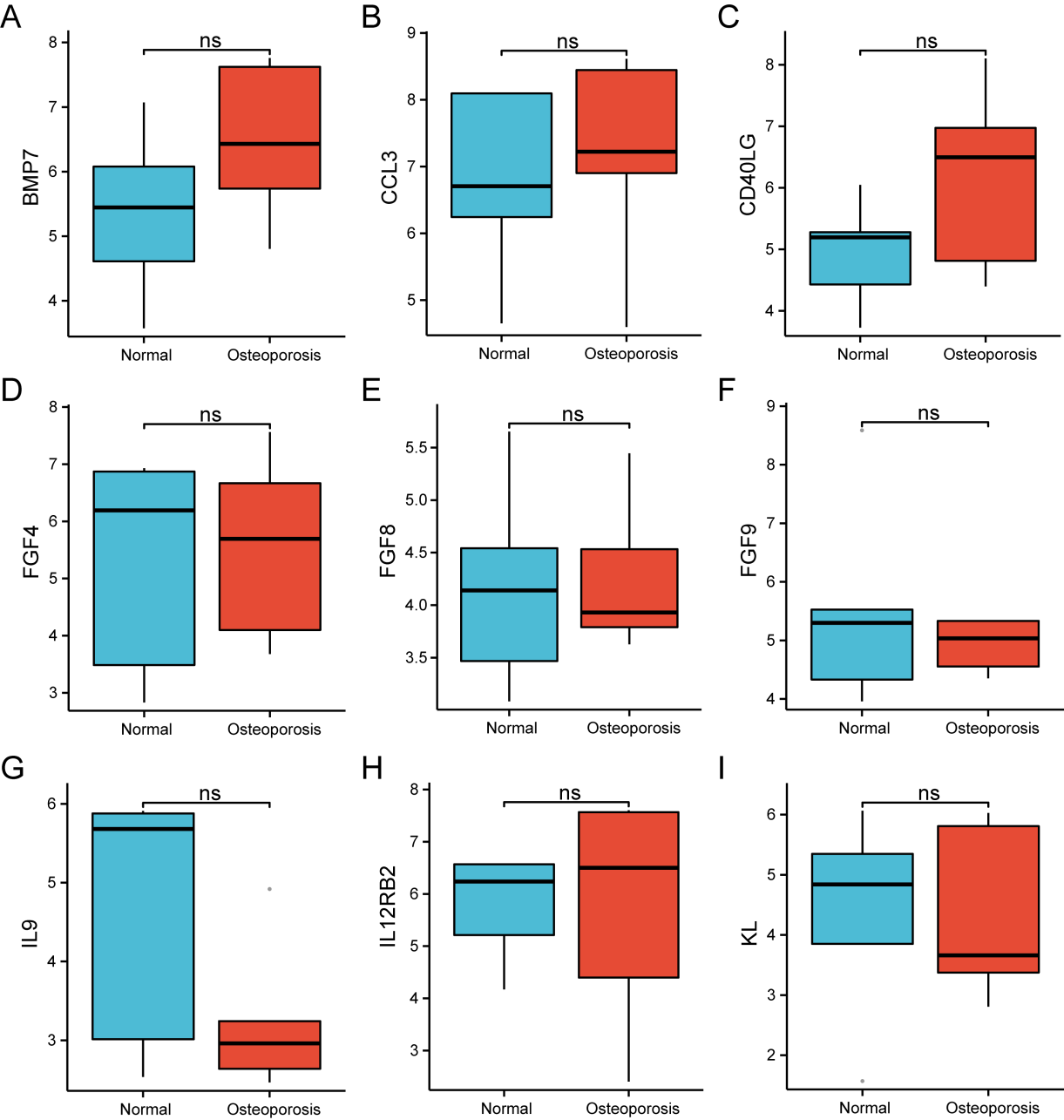


**Supplementary figure 2** Validation of hub gene expression in the GSE35959 dataset.


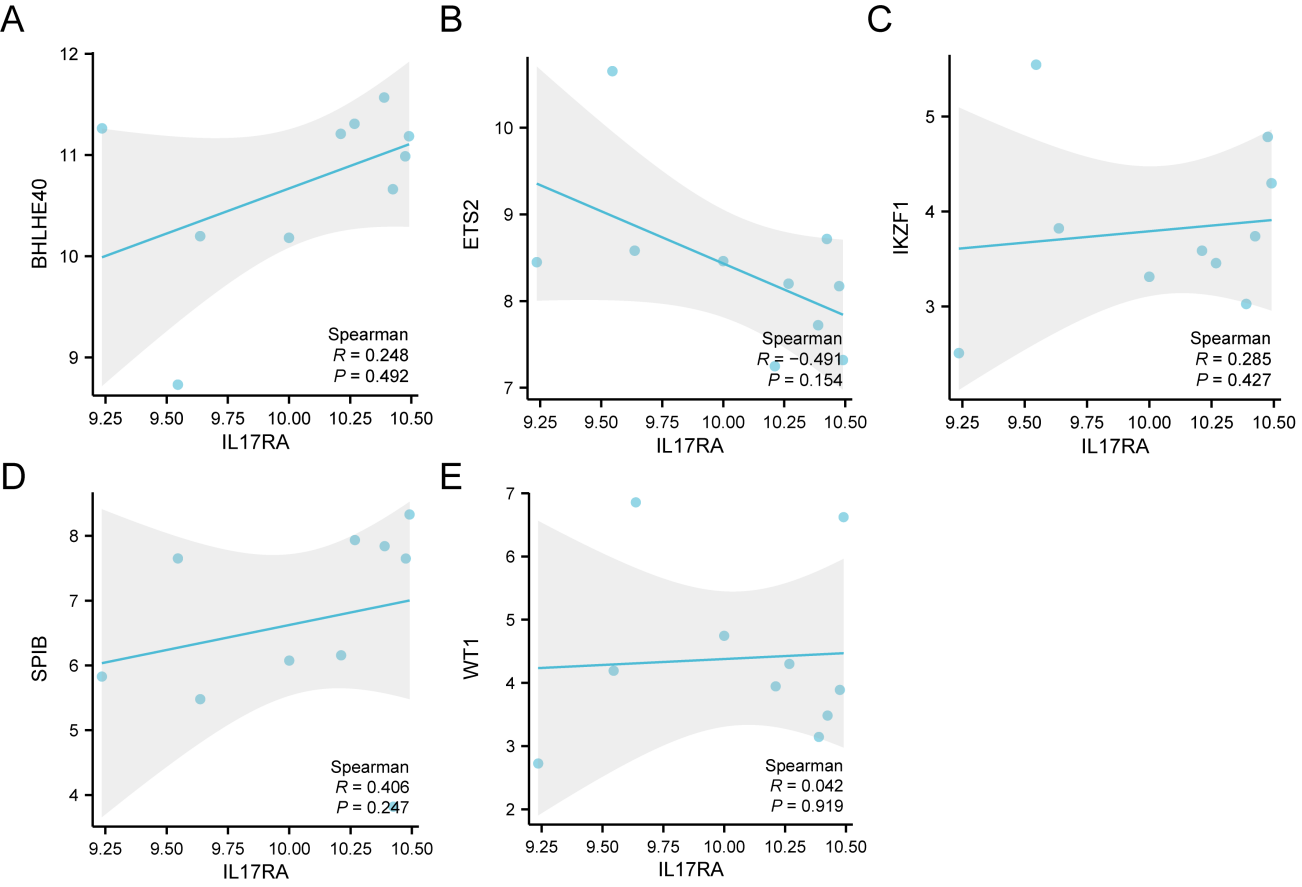


**Supplementary figure 3** Spearman correlation between *BHLHE40*, *ETS2*, *IKZF1*, *SPIB*, *WT1* and *IL17RA*.


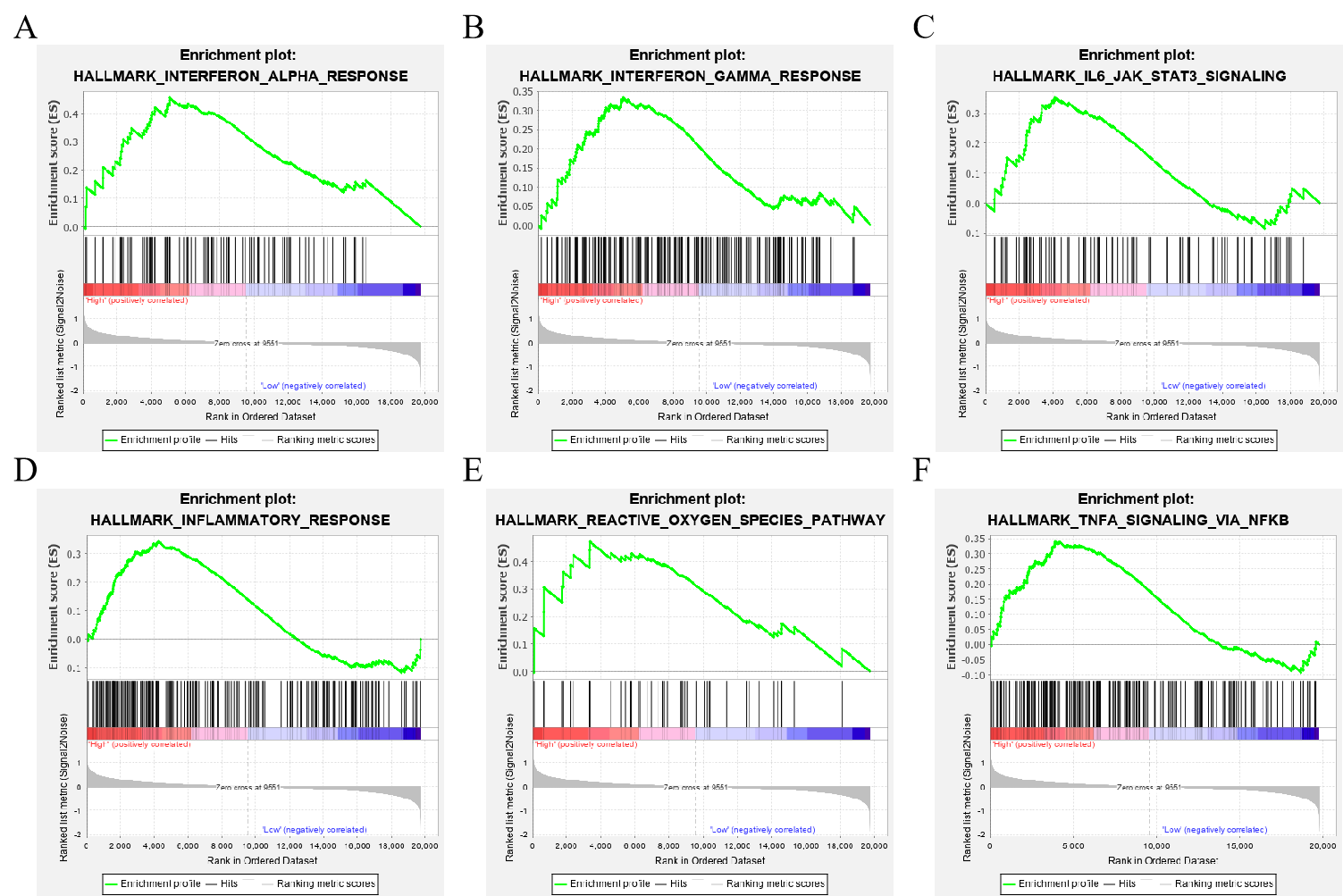


**Supplementary figure 4** Performing GSEA through the stratification of samples according to IL17RA expression.
